# Effect of Time Management Training on Anxiety, Depression, and Sleep Quality

**Published:** 2018-12

**Authors:** Ping WANG, Xiaochun WANG

**Affiliations:** 1. School of Management, Yangtze University, Jingzhou 434023, China; 2. School of Computer Science, Yangtze University, Jingzhou 434023, China

**Keywords:** Time management training, Anxiety, Depression, Sleep quality

## Abstract

**Background:**

Anxiety and depression seriously affect sleep quality and mental health of perimenopausal women. Time management training is of great value in relieving negative emotions and improving subjective well-being. This study aims to explore the effect of time management training on anxiety, depression, and sleep quality of perimenopausal women.

**Methods:**

From January 2018 to July 2018, 114 women with perimenopausal syndrome were randomly selected in Wuhan, Hubei Province of China as the objects of the experimental group (n=58) and the control group (n=56). The control group did not participate in any training in this study; time management training was conducted in the experimental group according to Getting Things Done, with a total of 12 training sessions over six months with two hours for each interval of two weeks. The effect of the intervention was evaluated before and after the experiment using relevant scales.

**Results:**

After the intervention, the Self-Rating Anxiety Scale (SAS) score and Self-Rating Depression Scale (SDS) score of the experimental group are lower than those of the control group (*P*<0.05). The Pittsburgh Sleep Quality Index (PSQI) score and Time Management Disposition Inventory (TMDI) score of the experimental group are higher than those of the control group (*P*<0.05). A positive correlation exists between the improvement in time management disposition and the improvement in anxiety, depression, and sleep quality (*P*<0.001).

**Conclusion:**

Time management training has a positive effect on improving anxiety, depression, sleep quality, and time management disposition of perimenopausal women.

## Introduction

An individual’s time management disposition is a multi-dimensional and multi-level trait of personality. It is the individual’s psychological and behavioral characteristic in the use of time, and it is composed of three dimensions, including time value (social and personal orientation), time control (goal setting, planning, priority, time allocation, and feedback), and time efficacy (time management efficacy and time management behavioral efficacy) (1). Studies showed that time management disposition is related to academic achievement and job performance (2), self-confidence, achievement motivation, psychological stressors, and personality (3). Perimenopause happens to women who are over 40 years old (4). From the perspective of physiological symptoms and time period, perimenopause is a less than one-year period after the last menstrual period of women who are close to menopause and suffer ovarian dysfunction (5). Perimenopause is an important turning point in women’s physiological development and indicates that women’s physiological development has entered a new stage. During this period, the strong fluctuations in women’s estrogen levels are likely to trigger the syndrome dominated by vegetative neurological disorders and accompanied by a series of psychotic symptoms, known as perimenopausal syndrome and “menopausal syndrome” (6).

One of the typical symptoms of perimenopausal syndrome is emotional symptoms. Owing to physical changes and endocrine disorders, women suffer from large mood swings during this period, and they tend to feel angry and experience intense anxiety. Strong depression and even severe depressive symptoms are also possible (7). Perimenopausal women had significantly higher levels of depression and anxiety than women in the general group, and such negative emotions such as anxiety and depression affected menopausal women’s sleep quality (8). More than 43% of women reported varying degrees of sleep disorders, and that these women also suffered emotional problems such as depression (9).

Complex causes of depression, anxiety, and sleep quality problems were observed in perimenopausal women, including both physiological and environmental factors (10). As the medical model shifts from simple biomedicine to the physiological–psychological–social medical model, the role of psychological factors increasingly attracts researchers’ attention (7). Research reported that the rapid economic and social development pose increasingly higher requirements on corporate employees, and the special nature of their work which should be finished within a limited time often leads to excessive burdens. As a result of greater psychological pressure, they became a high-risk group of mental illness (1). Therefore, perimenopausal women in enterprises, communities, and universities were involved in this study as the research objects to explore the factors that affect depression, anxiety, and sleep in this group from a psychological perspective and to use a targeted positive intervention. Therefore, improving the mental health level of perimenopausal women is of great significance.

Regarding the improvement in mental health problems such as depression and anxiety, time management disposition has been favored by researchers in recent years. Time management disposition reflects an individual’s attitude toward time and cognitive psychology in terms of time use as well as the individual’s time value and time-use behavior (1). Previous studies showed an important relationship between time management disposition, depression, and anxiety tendencies. Specifically, strengthening time management training will help reduce depression and anxiety levels (12). Moreover, research on the relationship between time management disposition and employees’ mental health revealed a significant negative correlation between employees’ time management disposition and each mental health factor, indicating that employees with higher mental management disposition had higher mental health levels (13). Patients with sleep disorders tended to have problems with time management disposition, especially in terms of time control and time efficacy (14). In summary, time management disposition may be negatively correlated with depression, anxiety, and sleep problems. Hence, the individual’s time management efficacy can be improved through time management training to reduce depression, anxiety, and sleep problems.

The innovation of this study lies in the application of time management training for the first time to improve mental health of perimenopausal women and the exploration of ways to deal with their emotional and sleep problems from the perspective of the psychological characteristics of perimenopausal women’s time management. On the other hand, the effect of time management training on emotional problems in perimenopausal women such as depression and anxiety as well as on their behavioral issues such as sleep quality problems was investigated in this study. Although emotions and behaviors were not comprehensively considered in previous studies, the relationship between emotional and behavioral problems in perimenopausal women is further examined in this study. This study provides a new intervention program to better improve mental health of perimenopausal women.

## Materials and Methods

From January 2018 to July 2018, 128 women with perimenopausal syndrome were randomly selected from three communities in Caidian District, Hongshan District, Hannan District, two large enterprises (Wuhan Branch of China Mobile and Dongfeng Motor Corporation), and health service stations in Wuhan, Hubei Province as the objects of the experiment. They were randomly divided into 64 members in the experimental group and 64 members in the control group. A total of 14 objects withdrew from the experiment, including six in the experimental group and eight in the control group. Finally, a total of 114 objects with 58 members in the experimental group and 56 members in the control group participated. Enrollment criteria: 1) those who participated in the experiment voluntarily, desired to change themselves, and signed the informed consent; 2) those who met Chinese Classification of Mental Disorders (CCMD-3) (15). Exclusion criteria: those with organic diseases, immune system diseases, blood system diseases, mental diseases, hormone replacement therapy, and long-term use of sedative hypnotic drugs. The general information of the two groups is shown in Table 1. Table 1 shows no significant difference between the two groups in age, education level, and occupation (P>0.05).

**Table 1:** Comparison of general information between experimental and control groups

***Variable***		***Experimental group (n=58)***	***Control group (n=56)***	***P***
Age(yr)		53.5±4.5	52.5±4.7	0.248
Housing area		89±7	91±9	0.187
Education level	Junior high school and below	25	23	0.690
	High or polytechnic school	18	16	
	College and above	15	17	
Marital status	Married	45	47	0.717
	Unmarried	4	3	
	Other	9	6	
Menstrual condition	Unmenopausal	25	28	0.267
	Menopausal	33	28	
Nationality	Han	54	53	1.000
	Other	4	3	
Employment situation	Unemployed	24	21	0.672
	Employed	34	35	
Whether lives alone	Alone	6	8	0.927
	With spouse	25	24	
	With children	21	19	
	Other	6	5	
Family economic situation	Poor	4	3	0.338
	Relatively poor	13	11	
	General	20	21	
	Relatively good	16	18	
	Quite good	5	8	

### Research tools

***1) Self-rating Anxiety Scale (SAS)***: Scoring Criterion: It includes 20 items and uses four-level scoring which is then converted to a full score of 100. A higher score means more apparent anxiety (16).
***2) Self-rating Depression Scale (SDS)***: Scoring criterion: It includes 20 items and uses four-level scoring which is then converted to a full score of 100. A higher score means more apparent depression. (17).
***3) Pittsburgh Sleep Quality Index (PSQI)***: Scoring criterion: The 18 self-report items participating in scoring are combined into seven components, namely, sleep quality, sleep latency, sleep duration, habitual sleep efficiency, use of sleeping medication, sleep disturbances, and day-time dysfunction. They are assessed using the 0–3 scoring method, and the total score is 21 points. A higher score means poorer sleep quality (18).
***4) Time Management Disposition Inventory (TMDI):*** Prepared by Huang et al. (19), it is composed of 44 items, including three subscales, which are time control observation scale, time value scale, and time efficacy scale. The test–retest reliability coefficient of each dimension of the scale is between 0.71 and 0.85; the internal consistency coefficient is between 0.58 and 0.83; the consistency coefficient of the three subscales is between 0.62 and 0.81. The scale has good content validity and construct validity.
***5) Getting Things Done (GTD):*** Based on the time management model (20) (collecting, organizing, reviewing, and executing), the time management notebook and GTD time management software on the market are combined with the realistic life status of the participants to develop self-edited GTD time management notes.

### Intervention method

***1) Control group***: The control group did not participate in any training in this study.
***2) Experimental group***: The experiment group was taught time management knowledge, practiced the GTD method, and finally formed three training feedback stages, including 12 training sessions over six months with two hours for each interval of two weeks. The first stage is the time management knowledge inculcation stage. The first task at this stage was to promote members to understand the value of time management disposition, to establish training objectives and group norms, and to achieve this through the first training. The second task was to teach relevant knowledge, the three dimensions of time management disposition, namely, time value, time control, and time efficacy, and the GTD method to achieve this through the second and third training. The second stage is the stage of time management practice. At this stage, practical management tasks were created based on the actual situation of the research objects to improve time management disposition of the research subjects to the largest extent in practice. In this stage, GTD notebooks were issued, and the objects were required to take electronic notes every day to help the objects better control their time. Taking notes not only allowed the objects to control their daily use and waste of time but also helped them reflect and find a way suitable for themselves. The counselor also discussed with the objects about their time management plan and implementation to obtain the best time management effect, which can be achieved through the 4th–11th training sessions. The third stage is the feedback stage. The GTD notes of the objects and their feelings about the activity were used to form feedbacks. The objects shared their learning experience with each other, reflected on the experience, and encouraged each other. The reflective feedback program was added to the 9th–12th training sessions. The effect of training was examined by measuring time management disposition before and after the experiment.

### Statistical methods

The data were analyzed by SPSS15.0 statistical software. The quantitative data were expressed as mean ± standard deviation. The comparison between two groups of means was conducted using independent sample t test. The inter-group comparison was conducted using paired t test. The qualitative data were represented by the number of cases. The inter-group comparison was conducted using chi-square test or rank-sum test. The correlation analysis between two variables was conducted using Spearman rank correlation analysis. The difference is statistically significant at P<0.05.

## Results

### General information of the research objects

The experimental groups had 64 objects, but 58 completed the intervention follow-up. The control group had 64 objects, but 56 completed the follow-up. The general information of the two groups is shown in Table 1. It shows that the general information of the two groups is comparable.

### Comparison of SAS before and after intervention

Table 2 shows no significant difference in the SAS score between the two groups before the intervention (t=1.004, *P*=0.318). After the intervention, the SAS scores of the two groups both decrease, and the differences are statistically significant with those before the intervention (*P*<0.001); the SAS score after the intervention and the decrease in the SAS score before and after the intervention show that the effect on the experimental group is greater than that on the control group, and the difference is statistically significant (*P*<0.05).

**Table 2:** Comparison of SAS before and after intervention

***Group***	***Before intervention***	***After intervention***	***Difference before and after intervention***	***P of inter-group comparison***
Experimental group (n=58)	59.6±7.9	50.9±8.8	8.7±6.5	<0.001
Control group (n=56)	61.2±9.1	45.7±8.1	15.5±7.0	<0.001
*t*	1.004	3.280	5.377	
*P*	0.318	0.001	<0.001	

### Comparison of SDS before and after intervention

Table 3 shows no significant difference in the SDS score between the two groups before the intervention (t=0.587, *P*=0.559). After three months of intervention, the SDS scores of the two groups both decrease, and the differences are statistically significant with those before the intervention (*P*<0.01); the SDS score after the intervention and the decrease in the SDS score before and after the intervention show that the effect on the experimental group is greater than that on the control group, and the difference is statistically significant (*P*<0.05).

**Table 3:** Comparison of SDS before and after intervention

***Group***	***Before intervention***	***After intervention***	***Difference before and after intervention***	***P of inter-group comparison***
Experimental group (n=58)	61.3±7.2	53.6±8.2	7.7±6.3	<0.001
Control group (n=56)	62.2±9.1	48.5±8.3	13.7±7.2	<0.001
*t*	0.587	3.300	4.740	
*P*	0.559	0.001	<0.001	

### Comparison of sleep quality before and after intervention

Table 4 shows no significant difference in the sleep quality score between the two groups before the intervention (t=0.841, *P*=0.402). After the intervention, the sleep quality scores of the two groups both decrease, and the differences are statistically significant with those before the intervention (*P*<0.01); the sleep quality score after the intervention and the decrease in the sleep quality score before and after the intervention show that the effect on the experimental group is greater than that on the control group, and the difference is statistically significant (*P*<0.05).

**Table 4:** Comparison of SDS before and after intervention

***Group***	***Before intervention***	***After intervention***	***Difference before and after intervention***	***P of inter-group comparison***
Experimental group (n=58)	12.6±2.9	8.9±2.8	3.7±1.5	<0.001
Control group (n=56)	12.2±2.1	10.7±2.2	1.5±1.0	<0.001
*t*	0.841	3.808	9.180	
*P*	0.402	<0.001	<0.001	

### Comparison of time management disposition before and after intervention

Table 5 shows no significant difference in the time management disposition score between the two groups before the intervention (t=0.751, *P*=0.454). After the intervention, the TMDI scores of the two groups both increase, and the difference of the experimental group with the pre-intervention value is statistically significant (*P*<0.001). The TMDI score of the control group increases slightly; however, no significant difference is noted when compared with the pre-intervention value (*P*=0.092); the time management disposition score after the intervention and the decrease in the time management disposition score before and after the intervention show that the effect on the experimental group is greater than that on the control group, and the difference is statistically significant (*P*<0.05).

**Table 5:** Comparison of time management disposition before and after intervention

***Group***	***Before intervention***	***After intervention***	***Difference before and after intervention***	***P of inter-group comparison***
Experimental group (n=58)	129.6±17.1	142.3±19.1	12.7±12.5	<0.001
Control group (n=56)	132.2±19.8	134.7±20.3	2.5±13.9	0.092
*t*	0.751	2.059	4.123	
*P*	0.454	0.042	<0.001	

### Correlation analysis of changes in time management disposition and changes in anxiety, depression, and sleep quality

The Spearman rank correlation analysis shows that the improvement in time management disposition after the intervention is positively correlated with the improvement in anxiety, depression, and sleep quality, thereby suggesting that good time management disposition is conductive to the improvement in anxiety, depression, and sleep quality, as shown in Table 6.

**Table 6:** Correlation analysis of changes in time management disposition and changes in anxiety, depression, and sleep quality

***Index***	***Correlation coefficient***	***P***
Anxiety	0.250	<0.001
Depression	0.278	<0.001
Sleep quality	0.314	<0.001

## Discussion

### Time management training can reduce anxiety levels of perimenopausal women

The analysis on the intervention results of this study (Table 2) shows no significant difference between the experimental group and the control group before the intervention (t=1.004, *P*=0.318), indicating that the two groups have the same levels before the intervention. After the intervention, the anxiety levels of the experimental group and the control group are significantly lower than those before the intervention (*P*<0.05), but the decrease in the anxiety level of the experimental group is quite significant after the intervention and more significant than the decrease in the anxiety level of the control group. The reasons can be analyzed as follows:

First, whether in the control or the experimental group, the anxiety level decreases significantly after the intervention. This phenomenon may be related to the occurrence and development of anxiety. The emergence of anxiety has causes in the interpretation of anxiety in the psychoanalytic school and the analysis of anxiety in humanistic psychology (21). That is, the causes of anxiety have a process of occurrence and development, and any cause has stages of growth and regression. Specific to this study, the experiment and the control groups experienced a certain degree of regression in the causes of anxiety with the progress of the intervention, thereby leading to the individual’s adaptation to the current state and reducing the level of anxiety. Marreta et al. (22) also showed in their study on perimenopausal women the different psychological stages in the entire perimenopausal period; and women’s self-perception and cognition were constantly changing at different stages, whereas physical and mental changes affected the level of anxiety.

Second, a significant decrease was observed in the anxiety level of the control group after the intervention, which is consistent with the results of Zhan et al. (23). In the study by Zhan et al., psychological intervention therapies including cognitive therapy, psychological support, music therapy, sleep anxiety, family support, and social support were used to intervene in 220 perimenopausal women. The results also demonstrate that the anxiety level decreases significantly not only in the experiment group but also in the control group. However, the study by Zhan et al. (23) had no in-depth analysis of the causes for the decline in anxiety of the control group. The causes for the significant decline in the control group which did not receive any psychological intervention include not only the natural development process of anxiety previously analyzed. In addition, the objects in the control group felt that they were still participating in the experiment and hoped to perform better owing to the social appreciating effect, thereby obscuring the true self and leading to the Hawthorne effect. However, owing to the lack of systematic time management training, the extent of the decline in anxiety of the control group is much lower than that of the experimental group.

Finally, the decrease in the anxiety level of the experimental group is quite apparent and better than that of the control group. This result again verifies the positive effect of time management training on reducing negative emotions. It is consistent with the research results of Fan et al. (12). However, in the study by Fan et al., the experimental group is a high-pressure group of women with a master’s degree. The consistency of the two findings fully demonstrates that perimenopausal women face higher pressure levels during this particular period, and previous studies showed that lowering pressure levels of high-anxiety people can contribute to the reduction of their anxiety (24–25). Therefore, the time management intervention in this study involves not only time management knowledge training but also the application of the GTD method, fully mobilizing the objects’ initiative and control of time utilization and effectively reducing anxiety of perimenopausal women.

### Time management training can reduce depression levels

The results of this study show that after the intervention training, the depression levels of the control group and the experiment group decrease significantly, but the decrease in the experimental group is higher than that in the control group, and the difference is statistically significant (*P*<0.05), suggesting a better effect. This finding is consistent with the results of Zhan et al. (23) who found that the depression levels of the control and the experiment groups decreased after the psychological intervention, but the effect on the control group is more apparent. The decline in sex hormone levels of perimenopausal women is an important factor leading to depressive symptoms, but this factor is clearly insufficient to explain the appearance of depressive symptoms (26). In the investigation of factors affecting perimenopausal women’s depressive symptoms, psychological factors are important factors which influence the emergence of depressive symptoms (27). The emergence of depression is often accompanied by a decrease in individual self-efficacy (28). Meanwhile, owing to physiological changes in perimenopausal women, physical changes reduce their self-efficacy to some extent. Jia et al. (29) conducted a five-week time management training for vocational students and found that after the intervention, their sense of time management self-efficacy improved, which in turn affected their general self-efficacy. In this study, the perimenopausal women were not only taught the knowledge of time management to improve their understanding of time use, but they were also required to observe and record their time use behavior in actual life to better improve their sense of time control. This can increase the rationality of time planning and effectiveness of time use among perimenopausal women, which in turn reduces the level of depression.

### Time management training can improve sleep quality

Sleep disorders are the most typical problem among perimenopausal women. Approximately 28%–64% of women were reported to have sleep disorders (30), and the occurrence of sleep disorders may also be caused by emotional problems such as depression and anxiety. A vicious circle could exist between them. The results of this study indicate that time management training can significantly improve sleep quality of the experimental group. Although the problem of sleep disorders in the control group is significantly lower than before the intervention, the effect is not as good as in the experimental group.

First, the increase in sleep quality of the control group may be associated with the natural decrease in the levels of anxiety and depression previously analyzed. When perimenopausal women enter the perimenopausal period, they have difficulty accepting physical changes and adopt no effective coping style, thereby leading to a decline in their mental health (31). Over time, perimenopausal women begin to adapt to the physiological transition and accept it psychologically. This natural adaptation process is the basic skill of biological organisms (32).

Second, the significant improvement in sleep quality of the experiment group is mainly caused by the positive effect of time management training. Through time management training, perimenopausal women not only reflect on their own time management behaviors during the intervention process but also increase the efficiency of their time use and their individual control over time, thereby improving their sleep quality. On the other hand, time management training effectively reduces the objects’ negative emotions such as depression and anxiety, which is also helpful for improving sleep quality.

### Time management training can improve time management disposition levels

The results of this study also reveal no significant difference in time management disposition between the experiment and the control groups before the intervention; the time management level of the control group does not increase significantly after the intervention, but the time management level of the experiment group after the intervention training increases significantly. This result illustrates two issues: First, the decline in depression and anxiety levels of the control group previously analyzed is caused by the changes in the level of time management propensity. Second, the level of time management disposition does not naturally increase with time but requires systematic training and intervention.

Time management disposition includes time value, time control, and time efficacy (19). Among them, time control and time efficacy have the greatest influence on individual behaviors. In this study, knowledge lectures can effectively improve the individual’s understanding and perception of time value. The GTD method can help individuals better monitor their own use and management of time to better supervise their own time behaviors (33). Feedback and experience learning in group counseling can also assist individuals to better improve their time use. Therefore, time management training in this study can be used to improve overall time management disposition of perimenopausal women. Nonetheless, owing to limited conditions, multiple sets of experiment groups were not used in the training program in this study. Although the results of this study show that the time management training program can effectively improve time management disposition of perimenopausal women, whether the program still has a consistent and stable effect on other populations is not known. The cross-population consistency of the training programs can be verified only by setting different experiment groups of objects according to different ages and genders.

## Conclusion

1) Time management training could significantly reduce the levels of anxiety and depression in perimenopausal women. 2) Time management training could improve sleep quality of perimenopausal women. 3) Time management training could significantly improve the time management disposition level of perimenopausal women. 4) Time management training has a significant positive predictive effect on sleep quality and a significant negative predictive effect on anxiety and depression.

## Ethical considerations

Ethical issues (Including plagiarism, Informed Consent, misconduct, data fabrication and/or falsification, double publication and/or submission, redundancy, etc.) have been completely observed by the authors.
